# A novel *ABCD1* gene mutation causes adrenomyeloneuropathy in a Chinese family

**DOI:** 10.1002/brb3.1416

**Published:** 2019-09-26

**Authors:** Chao Wang, Hongchao Liu, Bing Han, Hui Zhu, Jingyao Liu

**Affiliations:** ^1^ Department of Neurology The First Hospital of Jilin University Changchun China; ^2^ Department of Endocrinology The Second Hospital of Jilin University Changchun China

**Keywords:** ABCD1, adrenomyeloneuropathy, Chinese family, missense mutation, X‐linked

## Abstract

**Background:**

Adrenomyeloneuropathy (AMN) is a rare genetic disease. In this study, a case of AMN was uncovered in a Chinese family.

**Methods:**

Clinical manifestations were collected and observed through medical records, physical examination, laboratory tests, and magnetic resonance imaging (MRI). Generation sequencing of the *ABCD1* gene was performed, and the pedigree of the family was analyzed.

**Results:**

The proband suffered from adrenocortical insufficiency at 8 years old and presented with a slowly progressive gait disorder at 21 years old. Physical examination, laboratory tests, and MRI showed that he had adult‐onset AMN manifestations, including spasticity and hyperactive tendon reflexes with Hoffman and Babinski signs in the limbs, difficulty in performing the heel‐to‐shin test, hyperpigmentation, increased levels of adrenocorticotropic hormone and very long‐chain fatty acids, decreased levels of corticosteroid and serum gesterol, and salient atrophy of the cervical and thoracic spinal cord. DNA analysis revealed a missense variant, c.290A>C (p.His97Pro) in exon 1 of the *ABCD1* gene, in the proband. Sanger sequencing confirmed that the proband's mother was heterozygous for the same variant. The *ABCD1* gene mutation transmitted in an X‐linked inheritance manner.

**Conclusion:**

A novel missense mutation in the *ABCD1* gene was identified in a Chinese family, which caused an unusual manifestation of adult‐onset AMN. This discovery is beneficial for the genetic counseling of patients with X‐linked adrenoleukodystrophy.

## Graphical abstract

(a) We report a case of adrenomyeloneuropathy in a Chinese family. (b) DNA analysis revealed a missense variant c.290A>C (p.His97Pro) in exon 1 of the *ABCD1* gene in the proband.

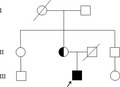

## 1 INTRODUCTION

X‐linked adrenoleukodystrophy (X‐ALD) is a metabolic disorder characterized by impaired oxidation of very long‐chain fatty acids (VLCFAs), which leads to the accumulation of VLCFAs in tissues, such as the brain white matter, spinal cord, and adrenal cortex. It is caused by mutations in the *ABCD1* gene, which codes for the peroxisomal transporter protein ALDP (Engelen et al., 2012). The *ABCD1* gene has been mapped to Xq28 (Migeon et al., 1981). More than 750 different variants in the *ABCD1* gene have been identified, and 343 are missense mutations (Kemp et al., 2001).

Adrenoleukodystrophy (ALD) can begin at different ages with a variety of manifestations, depending on the extent to which organs are affected (Moser et al., 1999). Among them, childhood cerebral ALD and adult‐onset adrenomyeloneuropathy (AMN) are the two main phenotypes of X‐linked ALD (Moser et al., 2000). Adult‐onset AMN usually begins at a later age and affects the spinal cord pyramidal tracts, dorsal columns, and peripheral nerves (Engelen, Kemp, & Poll‐The, 2014). Approximately 20% of AMN patients show cerebral demyelination in the late stage of the disease, occasionally with Wallerian degeneration of the corticospinal tract or cerebellar atrophy (de Beer, Engelen, & Geel, 2014).

Here, we present a Chinese family with adult‐onset AMN that was caused by a novel mutation in the *ABCD1* gene.

## 2 METHODS

### 2.1 Subjects

The present study was approved by the local Ethics Committee of Jilin University, Changchun, China. The proband and the proband's mother were studied after they signed a written informed consent form.

### 2.2 Magnetic resonance imaging (MRI)

The brain and spinal cord of the proband and the proband's mother were examined by MRI.

### 2.3 Identification of the *ABCD1* mutation

Blood specimens were obtained from the proband and the proband's mother, and the DNA was isolated. Generation sequencing was performed on ABI 9700 PCR and ABI 3730XL instruments (Life Technologies). The *ABCD1* mutation was identified through comparison with known human genome sequences and was defined as pathological mutation according to Human Gene Mutation Database (PMID: 27779215).

## 3 RESULTS

### 3.1 Clinical manifestations

**Figure 1:**
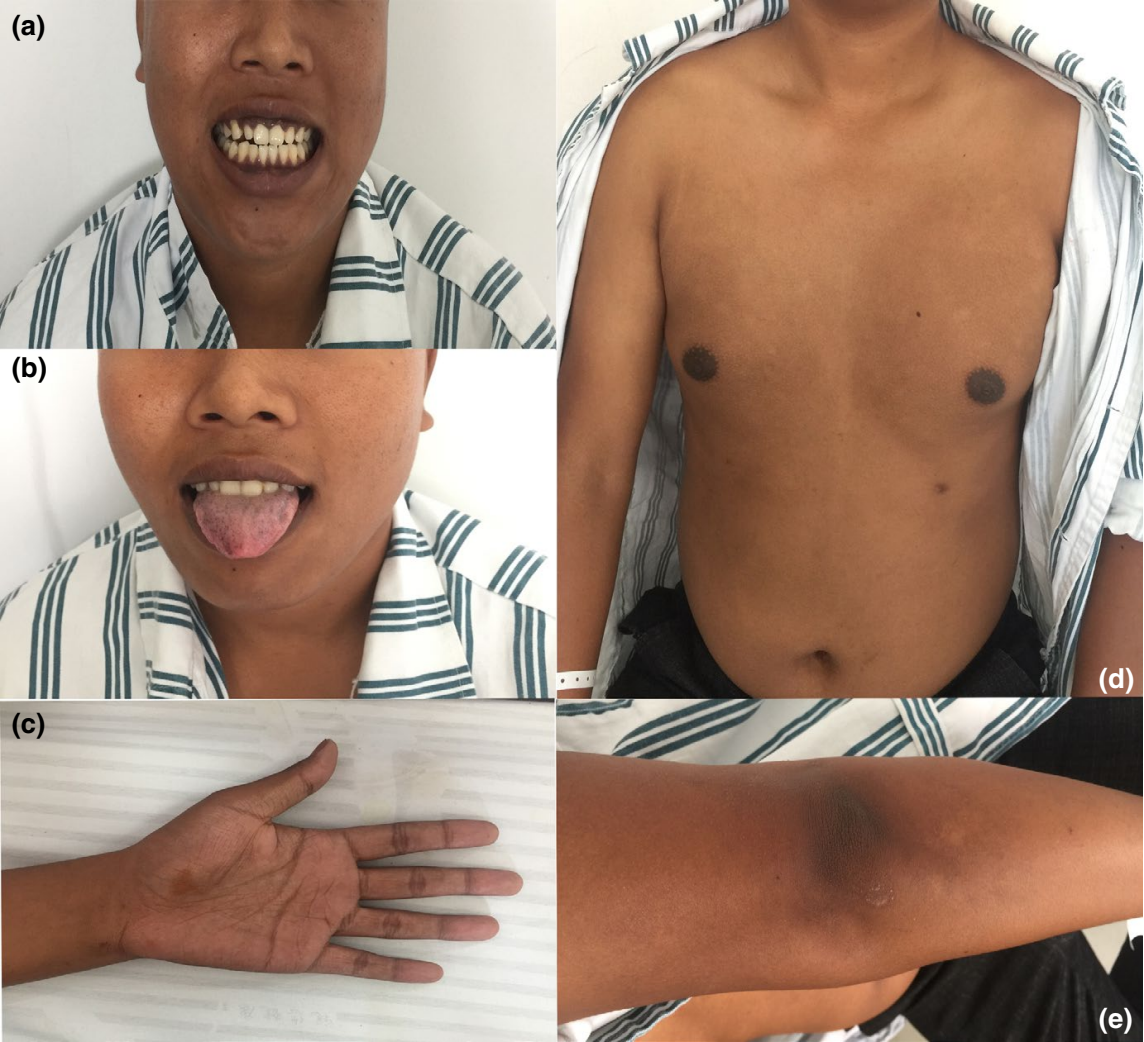
The patient showed hyperpigmentation in the gingiva (a), tongue (b), areolae (c), creases of the hand (d), and elbow joint (e)

The proband, a 21‐year‐old male college student, presented with a 1‐year history of slowly progressive gait disorder. He suffered from adrenocortical insufficiency when he was 8 years old. On neurological examination, spasticity and hyperactive tendon reflexes with Hoffman and Babinski signs in the limbs were observed. He was unable to perform the heel‐to‐shin test due to spasticity of the lower limbs, but he could still walk without assistance. He was alert without any intellectual disabilities. He could speak fluently and clearly without a detectable dysarthria. His hearing and vision were normal. There was no nystagmus or ophthalmoplegia. Both superficial and proprioception sensations were intact. Physical examinations showed hyperpigmentation, especially in the gingiva, tongue, areolae, creases of the hand, and elbow joint (Figure 1). The respiratory, cardiovascular, and abdominal examinations were unremarkable. The sphincter function was normal.

Laboratory investigations, including full blood counts, blood electrolytes, thyroid function, sex hormones, liver and renal function, and pituitary hormone levels (except gesterol), were normal. The adrenocorticotropic hormone (ACTH) levels were markedly increased (379.70, 440.40, and 288.40, normally 2.20–17.60 pM, at 0:00, 8:00, and 16:00, respectively), and the corticosteroid levels were decreased (<1 µg/dl, normally 1.40–6.30 µg/dl, at 0:00, 8:00, and 16:00). The serum gesterol level was decreased (0.11 ng/ml, normally 0.28–1.22 ng/ml). In addition, the VLCFAs were measured in the plasma. The hexadecanoic acid (C26:0) level was 2.519 μM (normally < 1.20 μM), the hexadecanoic acid and docosane acid ratio (C26:0/C22:0) was 0.06 μM (normally < 0.03 μM), and the tetradecanoic acid and docosane acid ratio (C24:0/C22:0) was 1.38 μM (normally < 0.62 μM).

**Figure 2:**
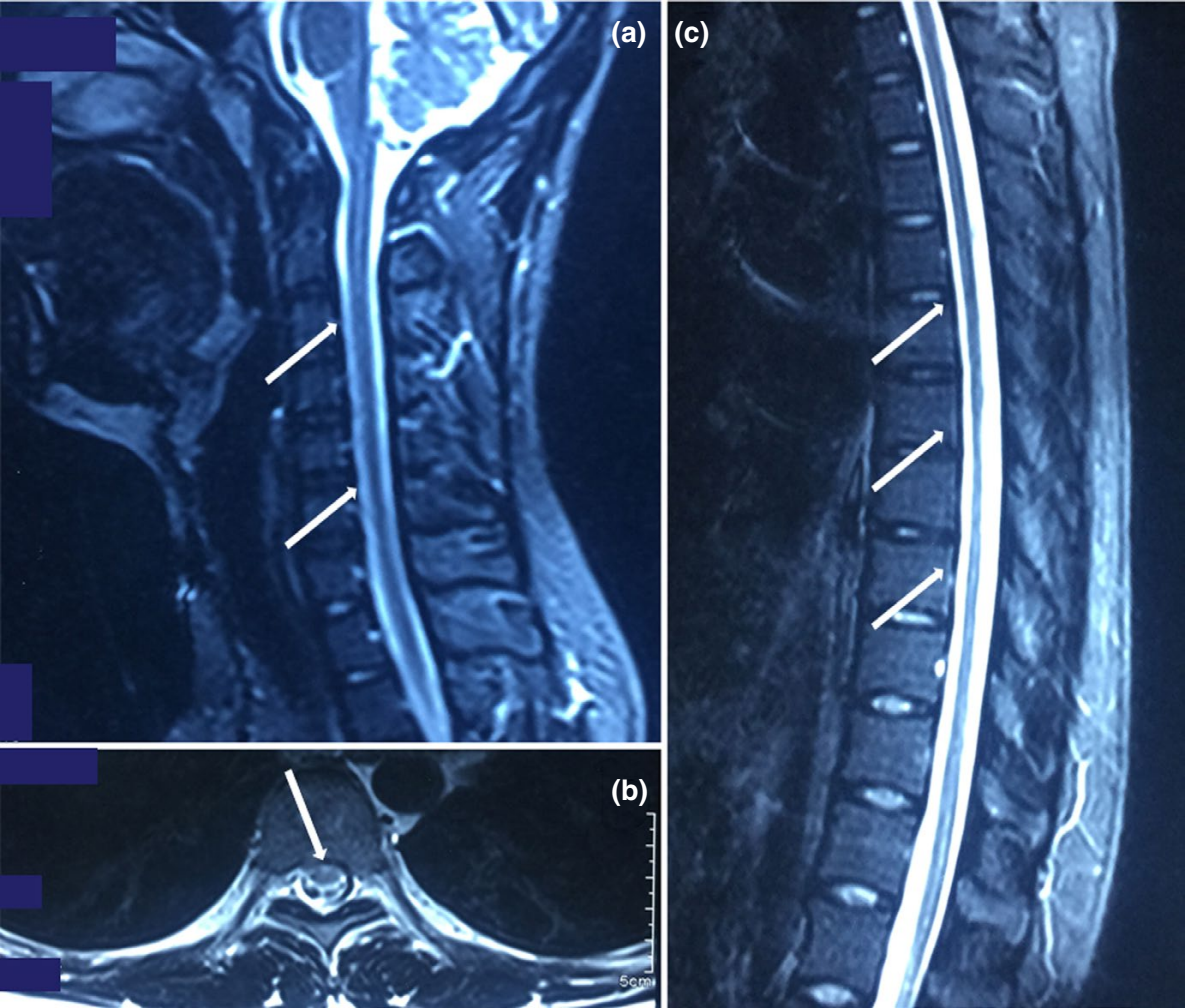
MRI analysis of the cervical and thoracic spinal cord of the proband. Severe spinal cord atrophy was detected. (a) Cervical cord T2‐weighted sagittal image; (b and c) thoracic cord T2‐weighted axial and sagittal image

Brain MRI was normal, and the spinal cord MRI showed salient atrophy of the cervical and thoracic regions (Figure 2), without any compression. Electrophysiological studies, including nerve conduction, visual evoked potentials, brainstem auditory evoked potentials, and somatosensory evoked potentials, did not reveal any abnormal findings. Based on these results, a diagnosis of X‐ALD was made.

### 3.2 Genetic analysis

**Figure 3:**
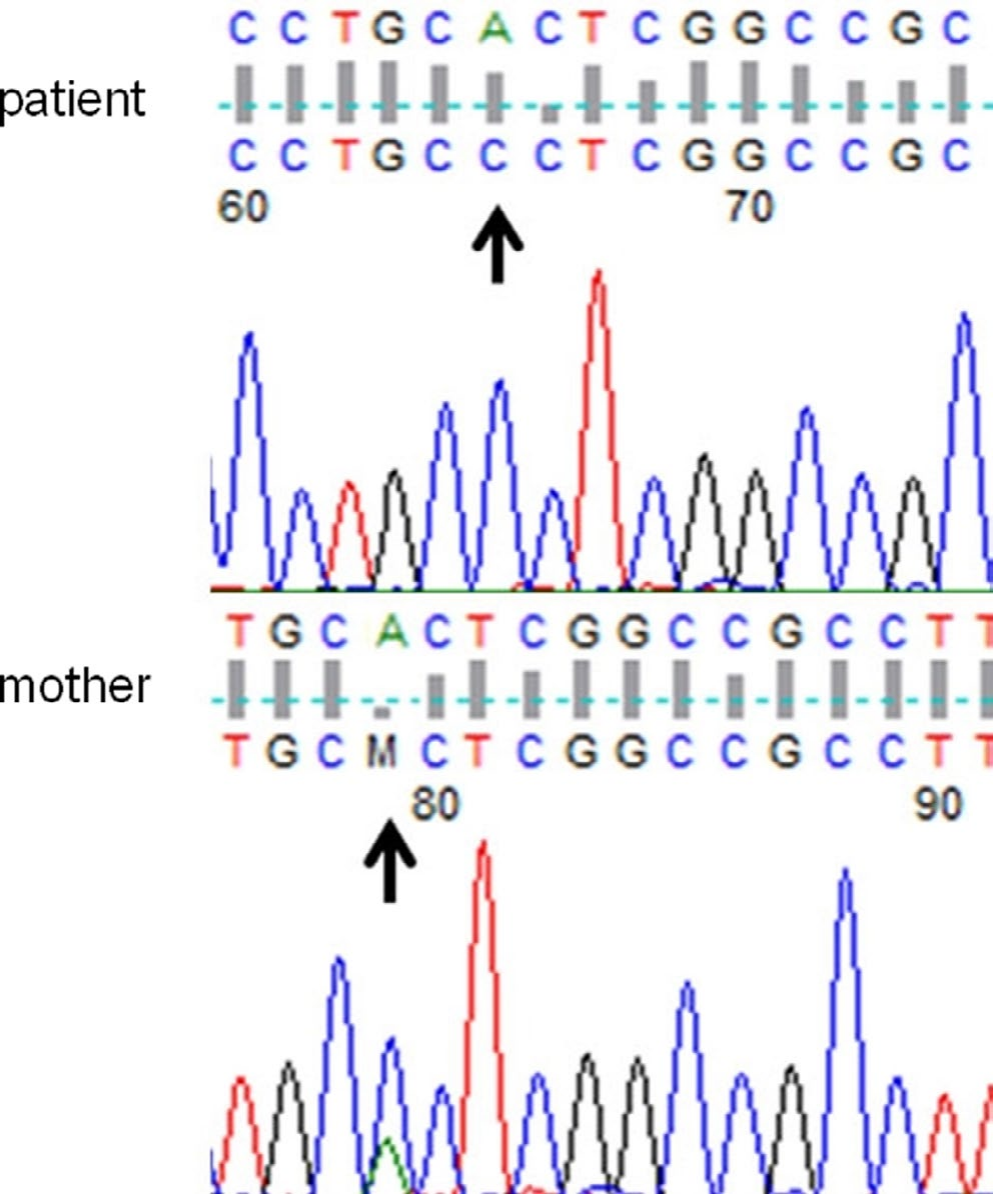
Sanger DNA sequencing profiles. The proband carried the novel mutation c.290A>C (p.His97Pro). The proband's mother was heterozygous for this mutation

**Figure 4:**
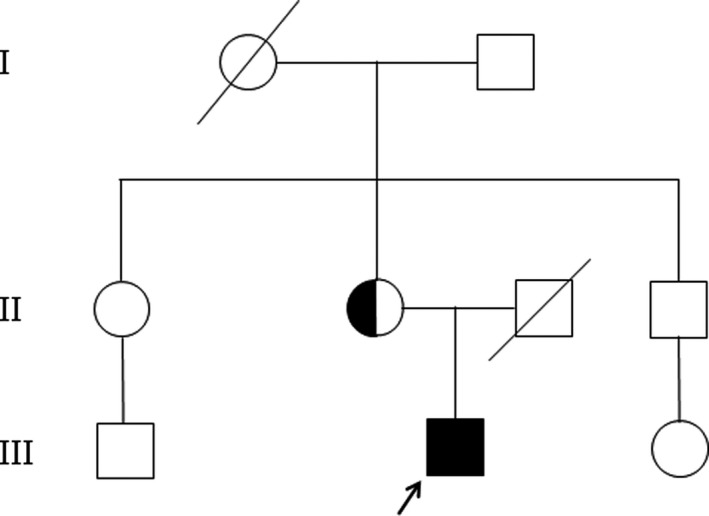
Pedigree of the Chinese family carrying the novel *ABCD1* mutation. , male; , female; , death; , male patient; , proband; , mutation carrier

Genetic analysis of the family members exhibited the missense mutation A>C at the 290th bp of the encoding region (c.290A>C), which led to a change in the 97th amino acid (from histidine to proline) in exon 1 of ABCD1 gene (Figure 3). Sanger sequencing confirmed the *ABCD1* point mutation in the proband. He was homozygous, and his mother was heterozygous for this variant. His father died several years ago; therefore, no blood sample could be obtained and tested. Based on the clinical manifestations and genotypic analysis results, a pedigree of the family with X‐ALD was constructed (Figure 4). The *ABCD1* point mutation followed an X‐linked inheritance pattern (Figure 4).

## 4 DISCUSSION

In the present study, the medical history, physical examination, laboratory tests, and MRI indicated that the proband had the clinical manifestations of ALD. Genetic analysis uncovered the missense mutation A>C at the 290th bp of the encoding region (c.290A>C) of the *ABCD1* gene, which was X‐linked. These results indicated that a point mutation in the *ABCD1* gene caused AMN in a Chinese family.

The proband was diagnosed with adrenocortical insufficiency at the age of 8 years, and he presented with neurological symptoms 12 years later. These symptoms are in agreement with the disease progression of X‐ALD, which is that adrenocortical insufficiency can be the presenting symptom in boys and men years or even decades before the onset of neurological symptoms (Hsieh & White, 2011) in as many as 35% of X‐ALD patients (Laureti et al., 1996). The proband had higher serum levels of ACTH and lower levels of corticosteroid; these levels are typical of approximately 50% of X‐ALD patients (Laureti et al., 1996). His decreased serum gesterol level indicated that he had the subclinical sign of testicular insufficiency, which is common among X‐ALD‐affected males (Assies, Gooren, Geel, & Barth, 1997). His higher serum ACTH level, lower corticosteroid level, and hyperpigmentation in the gingiva, tongue, areolae, creases of the hand, and elbow joint indicated that he had the “Addison‐only” phenotype of X‐ALD (van Geel, Assies, Wanders, & Barth, 1997). The proband became gradually symptomatic over a 1‐year period. After the initial clinical symptom of spastic gait disturbance started, he still completed his education and lived independently. Then, the symptoms of myelopathy, including spasticity, hyperactive tendon reflexes with Hoffman and Babinski signs in the limbs, and salient atrophy of the cervical and thoracic spinal cord without any compression, manifested. The relatively mild and slow progression of the disease in the proband was in contrast to childhood cerebral ALD, and it followed the pattern of adult‐onset AMN (Marino et al., 2007). Although the proband could still walk independently, it is likely that his disease will progress in 10–15 years with severe outcomes, such as wheelchair dependence (Qiu et al., 2018), progressive spastic paraparesis and sensory ataxia (Moser, Mahmood, & Raymond, 2007), and prominent spinal cord symptoms and axonopathy (Engelen et al., 2011; van Geel, Koelman, Barth, & Ongerboer de Visser, 1996), if he is not effectively treated. The MRI of the proband's brain appeared normal, with salient atrophy of the cervical and thoracic spinal cord and without any compression. There were no abnormalities in nerve conduction, visual evoked potentials, brainstem auditory evoked potentials, somatosensory evoked potentials, or cognition. It has been found that approximately 20% of AMN patients develop additional cerebral demyelination and moderate cognitive deficits over a period of 10 years (van Geel, Bezman, Loes, Moser, & Raymond, 2001). Therefore, the brain and spinal cord of AMN patients need to be carefully monitored by MRI. In summary, the clinical, electrophysiological, and radiological features of the proband were in accordance with the clinical manifestations of the AMN form of X‐linked ALD (Qiu et al., 2018).

In the present study, DNA analysis revealed the missense variant c.290A>C (p.His97Pro) in exon 1 of the *ABCD1* gene in the proband. The proband's mother was heterozygous for the same variant, while the proband's grandmother might also be heterozygous for the variant. The ALD symptoms in the male proband indicated that he had a mutated *ABCD1* allele on the X chromosome, which was confirmed by DNA sequencing. The transmission of the *ABCD1* mutation from his mother to the proband was also confirmed by the finding that his mother carried one allele of the *ABCD1* mutation and was heterozygous. Since the proband's mother did not present with any ALD manifestations, the *ABCD1* point mutation is recessive; it might have been transferred from the proband's grandmother to his mother. The *ABCD1* point mutation showed an X‐linked inheritance, which is expressed in most cases of X‐ALD (Kemp et al., 2001). The *ABCD1* gene encodes for the peroxisomal transporter protein ALDP (Engelen et al., 2012), which plays an important role in peroxisomal oxidation of VLCFAs (Engelen et al., 2012). Although some variations in the *ABCD1* gene, such as the “mis‐sense mutations” N13T and H97L, do not affect protein function or cause disease but rather represent polymorphisms (Morita et al., 2013), most disease‐causing mutations in the *ABCD1* gene cause loss of function of ALDP (Kemp et al., 2001), impairment of VLCFA oxidation, and accumulation of VLCFAs in tissues (Engelen et al., 2012). The higher levels of VLCFAs in the proband's plasma support that this point mutation in the *ABCD1* gene causes a deleterious change in ALDP (Wiesinger, Eichler, & Berger, 2015), loss of function of ALDP (Kemp et al., 2001), and impairment of VLCFA oxidation (Engelen et al., 2014, 2012).

In conclusion, a novel missense mutation in the *ABCD1* gene was identified, which caused an unusual manifestation of adult‐onset AMN in a Chinese family. These findings further increase our knowledge about *ABCD1* mutations and their associated phenotypes, which is beneficial for the genetic counseling of patients with X‐ALD.

## CONFLICT OF INTEREST

There are no conflicts of interest to disclose.

## AUTHOR CONTRIBUTIONS

Jingyao Liu carried out the molecular genetic studies, participated in the sequence alignment, and drafted the manuscript. Chao Wang and Hongchao Liu participated in the sequence alignment. Yanbo Hou participated in the design of the study. Hui Zhu conceived of the study, participated in its design and coordination, and helped to draft the manuscript. Chao Wang and Chunyu Dong wrote the paper. All authors read and approved the final manuscript.

## Data Availability

The data that support the findings of this study are available from the corresponding author upon reasonable request.

## References

[brb31416-bib-0001] Assies, J. , Gooren, L. J. , Van Geel, B. , & Barth, P. G. (1997). Signs of testicular insufficiency in adrenomyeloneuropathy and neurologically asymptomatic X‐linked adrenoleukodystrophy: A retrospective study. International Journal of Andrology, 20, 315–321. 10.1046/j.1365-2605.1997.00066.x 16130276

[brb31416-bib-0002] de Beer, M. , Engelen, M. , & van Geel, B. M. (2014). Frequent occurrence of cerebral demyelination in adrenomyeloneuropathy. Neurology, 83, 2227–2231. 10.1212/wnl.0000000000001074 25378668

[brb31416-bib-0003] Engelen, M. , Kemp, S. , de Visser, M. , van Geel, B. M. , Wanders, R. J. , Aubourg, P. , & Poll‐The, B. T. (2012). X‐linked adrenoleukodystrophy (X‐ALD): Clinical presentation and guidelines for diagnosis, follow‐up and management. Orphanet Journal of Rare Diseases, 7, 51 10.1186/1750-1172-7-51 22889154 PMC3503704

[brb31416-bib-0004] Engelen, M. , Kemp, S. , & Poll‐The, B. T. (2014). X‐linked adrenoleukodystrophy: Pathogenesis and treatment. Current Neurology and Neuroscience Reports, 14, 486 10.1007/s11910-014-0486-0 25115486

[brb31416-bib-0005] Engelen, M. , van der Kooi, A. J. , Kemp, S. , Wanders, R. J. , Sistermans, E. A. , Waterham, H. R. , … de Visser, M. (2011). X‐linked adrenomyeloneuropathy due to a novel missense mutation in the ABCD1 start codon presenting as demyelinating neuropathy. Journal of the Peripheral Nervous System, 16, 353–355. 10.1111/j.1529-8027.2011.00367.x 22176151

[brb31416-bib-0006] Hsieh, S. , & White, P. C. (2011). Presentation of primary adrenal insufficiency in childhood. Journal of Clinical Endocrinology and Metabolism, 96, E925–E928. 10.1210/jc.2011-0015 21470994

[brb31416-bib-0007] Kemp, S. , Pujol, A. , Waterham, H. R. , van Geel, B. M. , Boehm, C. D. , Raymond, G. V. , … Moser, H. W. (2001). ABCD1 mutations and the X‐linked adrenoleukodystrophy mutation database: Role in diagnosis and clinical correlations. Human Mutation, 18, 499–515. 10.1002/humu.1227 11748843

[brb31416-bib-0008] Laureti, S. , Casucci, G. , Santeusanio, F. , Angeletti, G. , Aubourg, P. , & Brunetti, P. (1996). X‐linked adrenoleukodystrophy is a frequent cause of idiopathic Addison's disease in young adult male patients. Journal of Clinical Endocrinology and Metabolism, 81, 470–474. 10.1210/jcem.81.2.8636252 8636252

[brb31416-bib-0009] Marino, S. , De Luca, M. , Dotti, M. T. , Stromillo, M. L. , Formichi, P. , Galluzzi, P. , … De Stefano, N. (2007). Prominent brain axonal damage and functional reorganization in "pure" adrenomyeloneuropathy. Neurology, 69, 1261–1269. 10.1212/01.wnl.0000276945.92950.69 17875914

[brb31416-bib-0010] Migeon, B. R. , Moser, H. W. , Moser, A. B. , Axelman, J. , Sillence, D. , & Norum, R. A. (1981). Adrenoleukodystrophy: Evidence for X linkage, inactivation, and selection favoring the mutant allele in heterozygous cells. Proceedings of the National Academy of Sciences of the USA, 78, 5066–5070. 10.1073/pnas.78.8.5066 6795626 PMC320333

[brb31416-bib-0011] Morita, M. , Kobayashi, J. , Yamazaki, K. , Kawaguchi, K. , Honda, A. , Sugai, K. , … Imanaka, T. (2013). A novel double mutation in the ABCD1 gene in a patient with X‐linked adrenoleukodystrophy: Analysis of the stability and function of the mutant ABCD1 protein. JIMD Reports, 10, 95–102. 10.1007/8904_2012_209 23430809 PMC3755581

[brb31416-bib-0012] Moser, A. B. , Kreiter, N. , Bezman, L. , Lu, S. , Raymond, G. V. , Naidu, S. , & Moser, H. W. (1999). Plasma very long chain fatty acids in 3,000 peroxisome disease patients and 29,000 controls. Annals of Neurology, 45, 100–110. 10.1002/1531-8249(199901)45:1<100:AID-ART16>3.0.CO;2-U 9894883

[brb31416-bib-0013] Moser, H. W. , Loes, D. J. , Melhem, E. R. , Raymond, G. V. , Bezman, L. , Cox, C. S. , & Lu, S. E. (2000). X‐Linked adrenoleukodystrophy: Overview and prognosis as a function of age and brain magnetic resonance imaging abnormality. A study involving 372 patients. Neuropediatrics, 31, 227–239. 10.1055/s-2000-9236 11204280

[brb31416-bib-0014] Moser, H. W. , Mahmood, A. , & Raymond, G. V. (2007). X‐linked adrenoleukodystrophy. Nature Clinical Practice Neurology, 3, 140–151. 10.1038/ncpneuro0421 17342190

[brb31416-bib-0015] Qiu, Y. , Xin, L. , Wang, Y. , Yu, Y. , Zou, K. , Zhou, Q. , … Hong, D. (2018). Novel ABCD1 gene mutation in adrenomyeloneuropathy with hypoplasia and agenesis of the corpus callosum. Neuro‐Degenerative Diseases, 18, 156–164. 10.1159/000490248 29966135

[brb31416-bib-0016] van Geel, B. M. , Assies, J. , Wanders, R. J. , & Barth, P. G. (1997). X linked adrenoleukodystrophy: Clinical presentation, diagnosis, and therapy. Journal of Neurology, Neurosurgery and Psychiatry, 63, 4–14. 10.1136/jnnp.63.1.4 PMC2169642 9221959

[brb31416-bib-0017] van Geel, B. M. , Bezman, L. , Loes, D. J. , Moser, H. W. , & Raymond, G. V. (2001). Evolution of phenotypes in adult male patients with X‐linked adrenoleukodystrophy. Annals of Neurology, 49, 186–194. 10.1002/1531-8249(20010201)49:2<186:AID-ANA38>3.0.CO;2-R 11220738

[brb31416-bib-0018] van Geel, B. M. , Koelman, J. H. , Barth, P. G. , & Ongerboer de Visser, B. W. (1996). Peripheral nerve abnormalities in adrenomyeloneuropathy: A clinical and electrodiagnostic study. Neurology, 46, 112–118. 10.1212/WNL.46.1.112 8559356

[brb31416-bib-0019] Wiesinger, C. , Eichler, F. S. , & Berger, J. (2015). The genetic landscape of X‐linked adrenoleukodystrophy: Inheritance, mutations, modifier genes, and diagnosis. The Application of Clinical Genetics, 8, 109–121. 10.2147/tacg.s49590 25999754 PMC4427263

